# Dermatofibrosarcoma protuberans following penetrating trauma: Case report

**DOI:** 10.1016/j.radcr.2026.06.099

**Published:** 2026-07-25

**Authors:** Ahmed Radu, Almahdi Abdulsalam, Salah Alzhari, Radwan Lagha, Ahmed Alasiddeeq

**Affiliations:** a Faculty of Medicine, University of Sabha, Sabha, Libya; b Faculty of Medicine, University of Benghazi, Benghazi, Libya

**Keywords:** Dermatofibrosarcoma protuberans, Soft tissue neoplasms, Magnetic resonance imaging, Sarcoma, Trauma, Wide local excision

## Abstract

Dermatofibrosarcoma protuberans is one of the rare types of cancer that originate from the dermal layer of the skin. It commonly affects the arms, legs, chest, shoulders, and trunk, but it can appear in any part of the body. We report a 43-year-old male presenting with a slow-growing mass on the back of the left shoulder that developed 1.5 years after a penetrating injury caused by an iron nail. Magnetic resonance imaging revealed evidence of a subcutaneous focal lesion at the back of the left shoulder, and wide local excision was performed, histopathological examination confirmed the diagnosis of dermatofibrosarcoma protuberans with tumor-free margins. The patient was followed up every 3 months, with no recurrence after 12 months of follow-up. This case underscores the possible link between trauma and dermatofibrosarcoma protuberans development, emphasizing the diagnostic challenges, management strategies, and the importance of long-term follow-up.

## Introduction

Dermatofibrosarcoma protuberans (DFSP) is a rare type of soft tissue sarcoma originating from the dermal layers of the skin. It accounts for approximately 6% of all sarcomas and less than 1% of all cancers [1]. It typically affects adults aged 20-50 years and most commonly involves the trunk, chest, legs, arms, and proximal extremities [2]. DFSP can develop after trauma or burn injuries, although the causal relationship remains uncertain [3,4]. MRI is the imaging modality of choice for the evaluation of DFSP because of its excellent soft-tissue contrast and multiplanar imaging capability. It plays a crucial role in determining tumor size, depth of invasion, and involvement of adjacent fascia, muscle, bone, and neurovascular structures, thereby facilitating preoperative planning. DFSP typically appears as an iso- to hypo-intense lesion on T1-weighted images, hyper-intense on T2-weighted images, and demonstrates marked contrast enhancement. These imaging characteristics help delineate tumor extent and guide surgical management. Extensive surgical excision with clear margins remains the cornerstone of treatment, while Mohs micrographic surgery (MMS) offers lower recurrence rates when available [5]. This report presents a case of DFSP associated with prior trauma and discusses the diagnostic and surgical management challenges in resource-limited settings, where access to advanced techniques like (MMS) is often limited. This case is unique because it documents a potential association between penetrating trauma and the development of DFSP, a sequence that is rarely reported in the literature.

## Case presentation

A 43-year-old male, with no medical or family history of tumors, presented with a mass at the site of a previous injury on the back of his left shoulder. The patient reported that the lesion first appeared approximately 1.5 years after a penetrating injury caused by an iron nail in the same area. He noted a period of accelerated growth in the 3 months preceding his presentation, which prompted him to request a medical evaluation.

On physical examination, there was a single palpable mass fixed to the underlying fascia, firm in consistency, measuring approximately 5 × 2 cm clinically. The overlying skin was intact without erythema, ulceration, or telangiectasia. No regional lymphadenopathy was detected.

MRI revealed a focal lesion at the posterior aspect of the left shoulder. The lesion was well defined within the subcutaneous tissues and measured approximately 5.3 × 2.3 cm. It demonstrated heterogeneous signal intensity, showing low signal intensity on T1-weighted images, intermediate signal intensity on T2-weighted images, and high signal intensity on STIR sequences. No evidence of invasion into the underlying muscular layer was identified, and the overlying skin remained intact (Fig. 1, Fig. 2, Fig. 3, Fig. 4, Fig. 5, Fig. 6). Based on these imaging characteristics, a soft tissue neoplasm such as DFSP was considered among the leading differential diagnoses (Fig. 7).

**Fig. 1:**
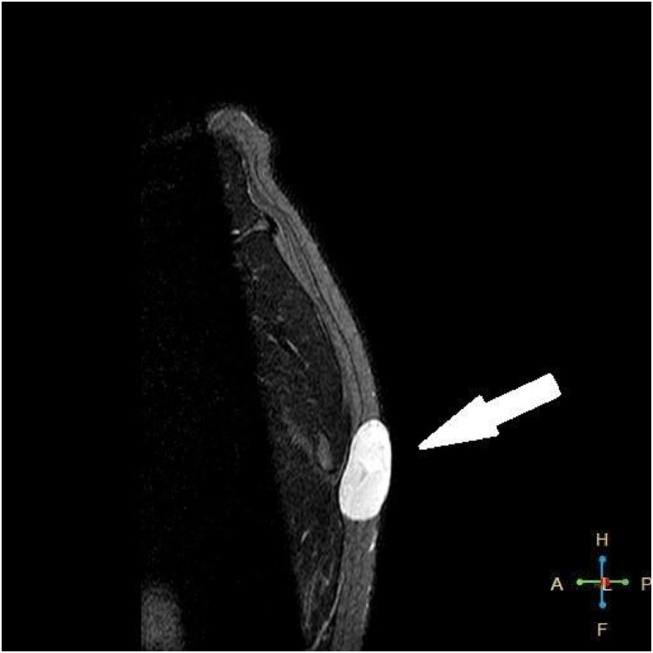
Sagittal fat-suppressed T2-weighted (or STIR) MRI scan showing a well-circumscribed, hyperintense (bright) subcutaneous mass in the posterior chest wall/back region (indicated by the white arrow).

**Fig. 2:**
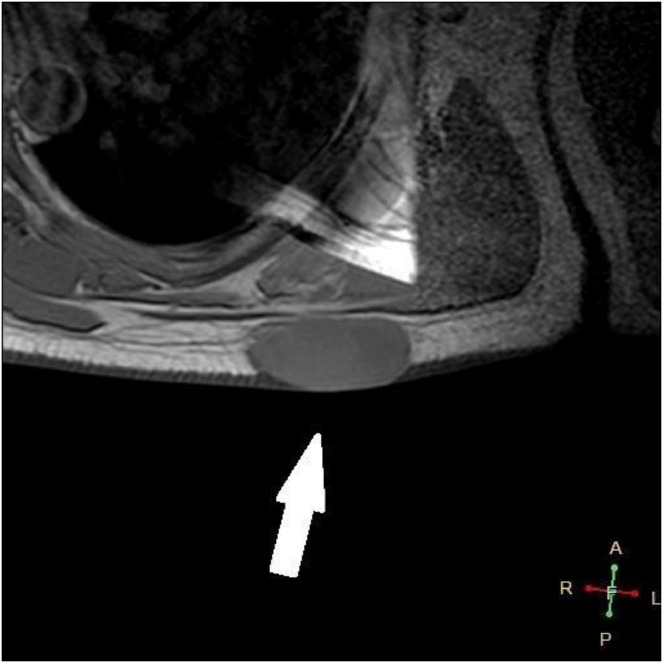
Axial T2-weighted MRI scan demonstrating a well-defined, hyperintense oval lesion located within the subcutaneous fat layer of the left posterior chest wall (indicated by the white arrow).

**Fig. 3:**
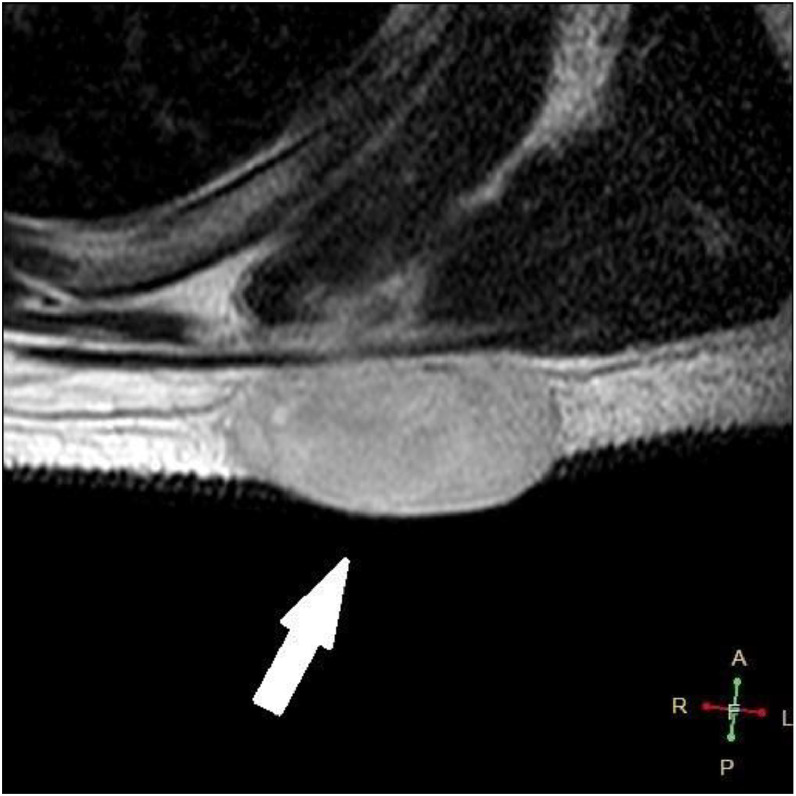
Close-up axial T1-weighted MRI scan showing an intermediate-to-low signal intensity, well-demarcated subcutaneous mass in the back/posterior thoracic wall (indicated by the white arrow).

**Fig. 4:**
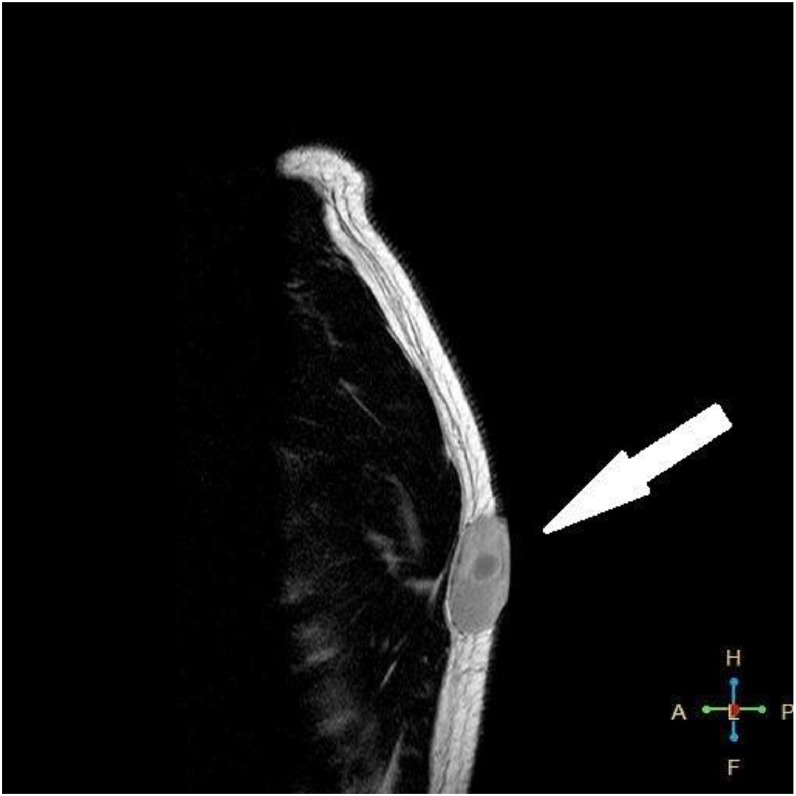
Sagittal T1-weighted MRI scan showing a well-defined, mildly hypointense to isointense subcutaneous mass along the posterior torso/back (indicated by the white arrow).

**Fig. 5:**
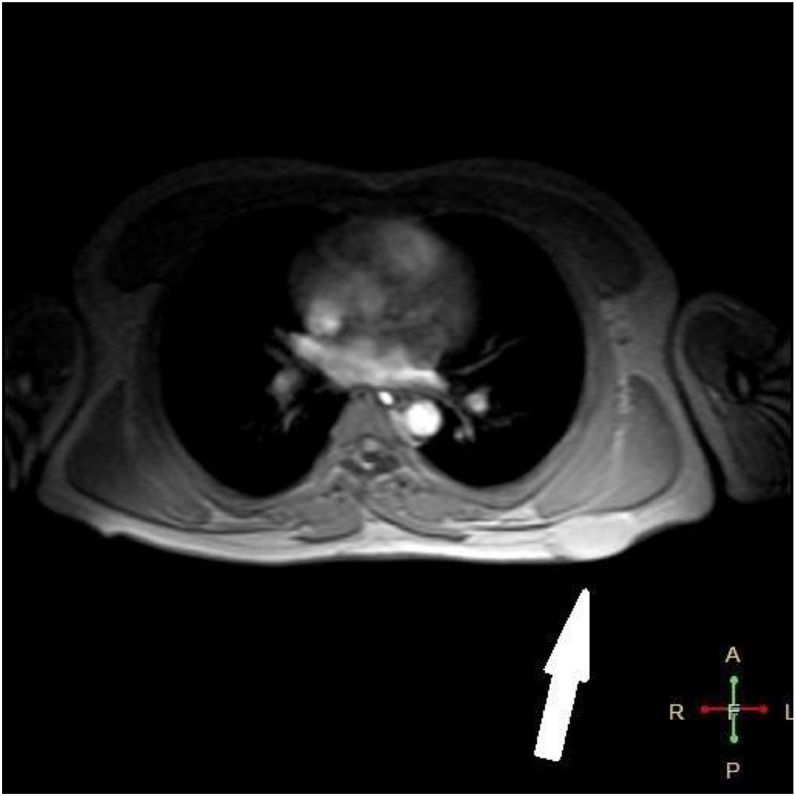
Axial contrast-enhanced (or fat-suppressed T1) chest MRI displaying a hyperintense, well-circumscribed subcutaneous lesion on the left side of the posterior chest wall (indicated by the white arrow).

**Fig. 6:**
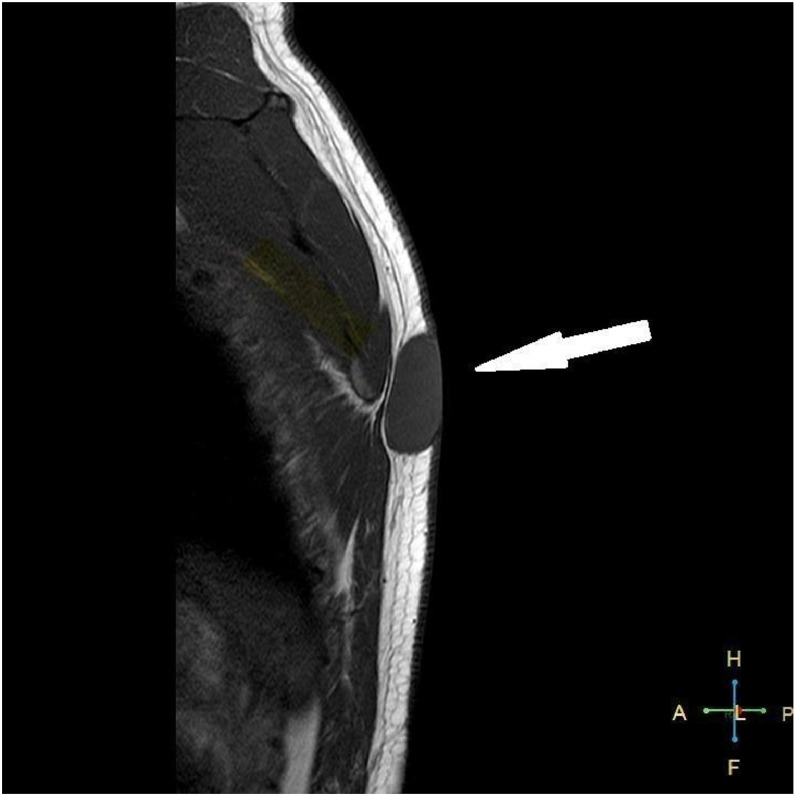
Sagittal contrast-enhanced T1-weighted MRI scan showing homogeneous enhancement of the subcutaneous mass in the posterior chest wall/back area (indicated by the white arrow).

**Fig. 7:**
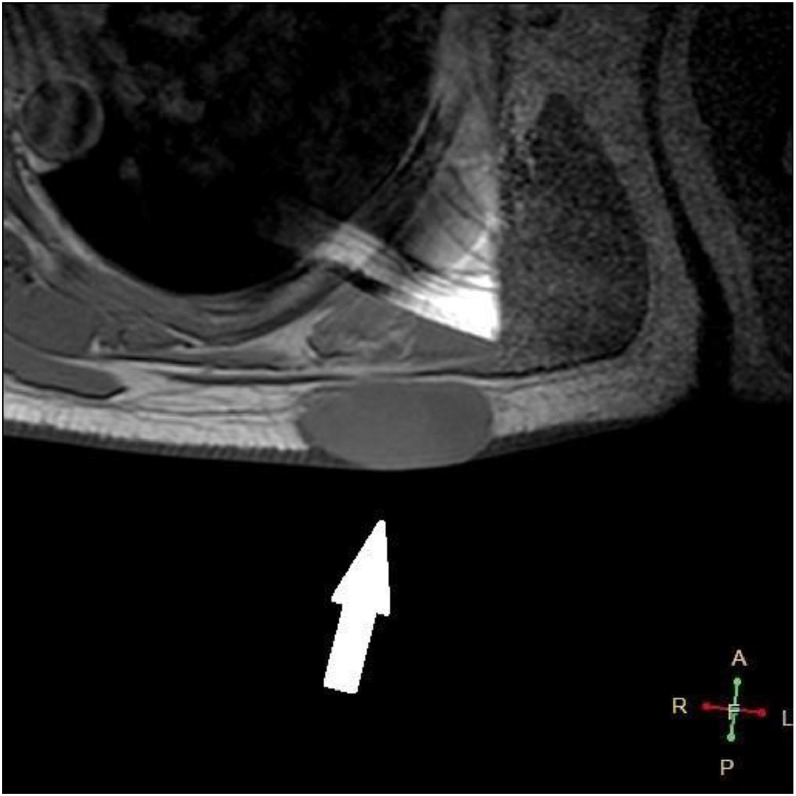
Axial fat-suppressed T1-weighted postcontrast MRI scan demonstrating the clear borders and uniform enhancement of the subcutaneous lesion on the left posterior chest wall (indicated by the white arrow).

Histopathological examination of the biopsy revealed a spindle-cell lesion composed of short fascicles of plump spindle cells arranged in a storiform pattern extending to subcutaneous fat with a honeycomb appearance, infrequent mitotic figures were seen. The immuno-histochemical analysis showed diffuse positive staining for CD34. Both morphological features and immuno-histochemical profile were consistent with DFSP.

The patient underwent wide local excision. The closest distance from the inferior surgical margin was 1 cm, while all remaining surgical margins exceeded 2 cm. The patient underwent regular clinical follow-up with ultrasonography and MRI at 3-month intervals. No evidence of local recurrence or distant metastasis was detected during the 12-month postoperative surveillance period. Informed consent was obtained from the patient.

## Discussion

DFSP represents an uncommon low to intermediate-grade sarcoma with a distinct clinical behavior. As illustrated in our case of a 43-year-old male with a trunk lesion, it typically affects middle-aged adults, with a well-established predilection for the trunk (50%), extremities (30%-40%), and head and neck (10%-15%) [6]. The pathogenesis of DFSP is driven by the characteristic chromosomal translocation t (17; 22) (q22; q13), which results in the COL1A1–PDGFB fusion gene and consequent PDGF-β–driven tumorigenesis [7]. The tumor in our patient appeared exactly where an iron nail had previously caused penetrating harm. This presentation supports the theory that localized wound-healing mechanisms and persistent inflammation may act as a trigger for tumor growth in genetically predisposed individuals, even though the direct relationship between trauma and DFSP remains unknown [8]. A possible connection between persistent inflammation and carcinogenesis has been suggested by similar trauma-associated DFSP cases documented in the literature [[8], [9]] (Table 1). Several well-documented cases show that DFSP develops at sites of prior trauma with different latency durations, as reported in Table 1. There are many parallels between our case and previous documented cases, especially when it comes to the tumor developing at the precise location of the penetrating injury. Our patient’s one-and half years latency time is within the broad range of 2-10 years documented in the literature. The idea that trauma may operate as a trigger for genetically predisposed people is further supported by the recurring pattern seen in these cases.

**Table 1:** Selected published trauma-associated DFSP cases compared with the present case.

Time from injury to symptoms appearance	Time from symptoms appearance to diagnosis	Type of trauma	Site of DFSP	Age/sex	Imaging findings	Study
12 y	2 mo	Keloid	Left shoulder	49/M	Not reported	Lee et al. [3]
3 y	2 y	Burn scar	Right flank region	32/M	Not reported	Agrawal et al. [4]
4 y	1 y	Blunt trauma	Mid-upper back	22/F	Not reported	Cabral et al. [9]
10 y	1 y	Keloid	Left shoulder	31/F	Not reported	Ward et al. [10]
1.5 y	3 mo	Penetrating nail injury	Left shoulder	43/M	MRI: Well-defined subcutaneous lesion, low T1, intermediate T2, high STIR, and no muscle invasion	Present case

Imaging findings were unavailable in the previously published reports except for the present case.

MRI was particularly valuable in the present case because it enabled accurate assessment of tumor extent and its relationship to adjacent structures, which is essential for surgical planning. The lesion demonstrated low signal intensity on T1-weighted images, intermediate signal intensity on T2-weighted images, and high signal intensity on STIR sequences. These findings are generally consistent with the MRI appearance of soft tissue sarcomas, which commonly exhibit low-to-intermediate signal intensity on T1-weighted imaging and intermediate-to-high signal intensity on T2-weighted imaging owing to their cellular composition and water content [11].

In addition to local staging, MRI contributed to the differential diagnosis. Post-traumatic lesions such as keloids may demonstrate variable enhancement patterns and are usually confined to the dermis and subcutaneous tissues. Fibromas typically exhibit lower T2 signal intensity because of their dense collagen content. In contrast, the progressive growth pattern, lesion size, and MRI characteristics observed in our patient were more suggestive of a soft tissue neoplasm such as DFSP. Furthermore, MRI excluded invasion of the underlying musculature and confirmed preservation of the overlying skin, thereby facilitating surgical planning and achievement of adequate resection margins [12].

The clinical size of the lesion was approximately 5 × 2 cm, while MRI demonstrated a lesion measuring 5.3 × 2.3 cm. The close correlation between clinical and radiological measurements further supported accurate preoperative assessment.

Clinically, DFSP usually manifests as a slow-growing, asymptomatic plaque or nodule, and our patient's slow-growing mass eventually showed signs of accelerated growth. This clinical development underscores the importance of examining any changing skin lesion as soon as possible. In post-traumatic cases, keloids, cutaneous fibromas, and other spindle cell tumors are the main differential diagnoses [10]. In this case, MRI was essential for surgical planning, as it showed the full extent and deep impact of the lesion. Complete surgical excision continues to be the cornerstone of DFSP therapy. Current guidelines recommend wide local excision with 2-3 cm margins [5,13]. Our achievement of consistent 2-3 cm margins in all directions, including the deep plane, demonstrates that it is feasible to follow recognized oncological principles even in resource-constrained contexts. The consistent margins throughout the excision, particularly in the challenging anatomical site of the upper back, underscore the importance of surgical expertise in treating DFSP in situations when access to Mohs micrographic surgery is limited. This demonstrates that meticulous planning and surgical practice can achieve optimal outcomes even without advanced instruments. The patient's lack of relapse after 12 months of follow-up, and the adequate surgical margins achieved, prove the effectiveness of this strategy. However, given the known recurrence potential of DFSP, continuous, long-term monitoring is still necessary to detect late relapses. This case highlights the need for clinicians to increase clinical awareness of DFSP in areas with limited diagnostic resources. Our findings highlight the importance of using DFSP in the differential diagnosis of any persistent or growing post-traumatic skin lesion. To ensure prompt diagnosis and appropriate surgical care, any solid, persistent, or growing mass at a site of previous trauma should raise suspicion of DFSP and require appropriate radiographic and histological evaluation. This report describes a single case in a male patient. The influence of sex and gender on the potential association between trauma and DFSP development could not be explored and should be investigated in larger, more diverse populations.

## Conclusion

This case summarizes a potentially compelling link between penetrating trauma and the development of DFSP, and also successfully demonstrated that extensive local resection with clear margins can achieve excellent tumor outcomes in resource-limited settings. A multimodal approach involving clinical assessment, characteristic MRI findings, and histological confirmation using the CD34 immuno-histochemical stain was used to diagnose the disease. MRI played a crucial role in lesion characterization, local staging, differential diagnosis, and surgical planning. The close correlation between clinical and radiological findings contributed to successful surgical management and achievement of tumor-free margins. Achieving consistently 2 cm margins, even at the difficult deep level, highlights that when performed by skilled surgeons, adherence to accepted surgical guidelines allows for the best DFSP care with conventional surgery. Physicians working in similar healthcare settings can greatly benefit from this experience.

## Declaration of generative AI and AI-assisted technologies in the writing process

During the preparation of this work, the author (Ahmed Radu) used ChatGPT and DeepSeek in order to assist with language editing and improving the clarity of the manuscript. After using these tools, the author reviewed and edited the content as needed and takes full responsibility for the content of the published article.

## Patient consent

Written informed consent was obtained from the patient for publication of this case report and accompanying images. A copy of the written consent is available for review by the Editor-in-Chief of this journal.
